# Automated Landslide-Risk Prediction Using Web GIS and Machine Learning Models

**DOI:** 10.3390/s21134620

**Published:** 2021-07-05

**Authors:** Naruephorn Tengtrairat, Wai Lok Woo, Phetcharat Parathai, Chuchoke Aryupong, Peerapong Jitsangiam, Damrongsak Rinchumphu

**Affiliations:** 1 School of Software Engineering, Payap University, Chiang Mai 50000, Thailand; naruephorn_t@payap.ac.th (N.T.); phetcharat@payap.ac.th (P.P.); 2 Department of Computer and Information Sciences, Northumbria University, Newcastle upon Tyne NE1 8ST, UK; 3 Center of Excellence for Natural Disaster Management (CENDIM), Chiang Mai University, Chiang Mai 50200, Thailand; chuchoke.a@cmu.ac.th (C.A.); peerapong@eng.cmu.ac.th (P.J.); damrongsak.r@cmu.ac.th (D.R.); 4 Department of Civil Engineering, Faculty of Engineering, Chiang Mai University, Chiang Mai 50200, Thailand

**Keywords:** landslide risk prediction, geographic information system, google map, machine learning, linear regression, artificial intelligence, long short-term memory

## Abstract

Spatial susceptible landslide prediction is the one of the most challenging research areas which essentially concerns the safety of inhabitants. The novel geographic information web (GIW) application is proposed for dynamically predicting landslide risk in Chiang Rai, Thailand. The automated GIW system is coordinated between machine learning technologies, web technologies, and application programming interfaces (APIs). The new bidirectional long short-term memory (Bi-LSTM) algorithm is presented to forecast landslides. The proposed algorithm consists of 3 major steps, the first of which is the construction of a landslide dataset by using Quantum GIS (QGIS). The second step is to generate the landslide-risk model based on machine learning approaches. Finally, the automated landslide-risk visualization illustrates the likelihood of landslide via Google Maps on the website. Four static factors are considered for landslide-risk prediction, namely, land cover, soil properties, elevation and slope, and a single dynamic factor i.e., precipitation. Data are collected to construct a geospatial landslide database which comprises three historical landslide locations—Phu Chifa at Thoeng District, Ban Pha Duea at Mae Salong Nai, and Mai Salong Nok in Mae Fa Luang District, Chiang Rai, Thailand. Data collection is achieved using QGIS software to interpolate contour, elevation, slope degree and land cover from the Google satellite images, aerial and site survey photographs while the physiographic and rock type are on-site surveyed by experts. The state-of-the-art machine learning models have been trained i.e., linear regression (LR), artificial neural network (ANN), LSTM, and Bi-LSTM. Ablation studies have been conducted to determine the optimal parameters setting for each model. An enhancement method based on two-stage classifications has been presented to improve the landslide prediction of LSTM and Bi-LSTM models. The landslide-risk prediction performances of these models are subsequently evaluated using real-time dataset and it is shown that Bi-LSTM with Random Forest (Bi-LSTM-RF) yields the best prediction performance. Bi-LSTM-RF model has improved the landslide-risk predicting performance over LR, ANNs, LSTM, and Bi-LSTM in terms of the area under the receiver characteristic operator (AUC) scores by 0.42, 0.27, 0.46, and 0.47, respectively. Finally, an automated web GIS has been developed and it consists of software components including the trained models, rainfall API, Google API, and geodatabase. All components have been interfaced together via JavaScript and Node.js tool.

## 1. Introduction

Landslides are a natural disaster where land or rocks can follow along the slopes of the mountain. In most cases, landslides occur with or after heavy rains, causing the soil to become so wet that the weight of the soil mass increases and as a result, the adhesion force between the soil masses decreases [1,2,3]. Landslides occur slowly or suddenly. If there are inhabitants or properties in the direction of soil flow, then they may also cause damage to life and property [4]. According to the statistics on landslides in Thailand, most of them occur in the mountainous regions from the upper central to the northern regions. Additionally, there is a southern region in which they sometimes occur. While not as frequent as floods, over the past 30 years, landslides have killed more than 500 people. Analysis results from the Department of Mineral Resources found that Thailand has 54 provinces or 1084 sub-districts affected by landslides. Areas at risk from landslides have been estimated by using various geospatial data to be analyzed together according to GIS techniques such as topographic data, area slope, geological data, information on the location of community sources, and land use information, etc.

The landslide occurrence is a complex process which is a combination of multiple interacting factors. In previous decades, traditional methods for landslide susceptibility assessment have relied on statistical-based approaches and data-driven approaches. For example, frequency ratio and index of entropy models [5,6], a logistic regression (LR) approach [7,8,9], and support vector machine (SVM) [10,11,12,13] are powerful approaches to predict potential landslides. However, these approaches require a large number of samples in order to yield high predicting accuracy. Landslides occur from time to time in random locations, but will significantly cause damage and impact on people and property. Disadvantages of traditional landslide prediction include: (1) the models generally require a large amount of prior knowledge and assumptions. (2) The networks are not sufficient to characterize the underlying non-linear features of landslides. (3) The networks are not sufficiently wide to consider the correlations between sub-regions. (4) The models encounter problems, such as over-fitting, time-consuming computation, ease of falling into local optima, and sensitivity to missing data, which affect the accuracy of prediction.

Recently, model-based learning approaches have been extensively exploited for landslide prediction. Artificial neural network (ANN) [14,15,16,17], long short-term memory (LSTM), and deep learning methods have been trained for learning landslide patterns and extracting crucial factors of landslide susceptibility. LSTM applies gate control logics and memory to learn long temporal dependency data in time series predictions [18,19,20,21]. For example, the research in [18] provided 14 factors which can be categorized into 3 groups. These are topographic, land cover, and hydrological factors, achieved by obtaining information from remote sensing images and a geographic information system. Historical landslides areas of study have a total of 369 landslides from 1970 to 2012. Machine learning was built by using LSTM with conditional random field. This method yields better landslide-prediction rate than multilayer perception, logistic regression, and decision tree. The dynamic model in [19] was proposed for foretelling the movement associated with the landslide in China in September 2015. The landslide area is about 200 × 300 m. The LSTM model is given by multiple factors of geological conditions, rainfall intensity, and human activities. Compared with a traditional mechanical model, the deep-learning-based models are able to extract inherent and deep features given by smaller data. Hence, the model-learning approach has good characteristics of dynamic feature to capture landslide pattern from a limited dataset.

Thailand is a country in the center of Southeast Asia which has a total size of 513,120 km^2^ (198,120 mi^2^). Most of Thailand’s terrain is the high mountainous area in the North, an upland plateau in the Northeast, a central plain in the middle and the sea in the South. Thailand’s climate is divided into three seasons which are the rainy, winter, and summer seasons. Large landslides have occurred in Thailand every 3–5 years [22]. Research on landslides and landslide forecasts in Thailand have constantly been developed. Various prediction approaches have been employed for estimating landslides risk such as the GIS, remote sensing techniques, statistical methods, or machine learning models. Phetchabun Province used GIS and remote sensing techniques in 2006 [23]. In 2010, a weighting factor and geotechnical methods were used to estimate the safety factor scores, then generate a landslide susceptibility map of Phuket Island by using APIs. Some studies have used statistical methods to predict landslide probability levels. A landslide probability model under multiple global climate models (GCMs) was applied to project the probability of landslide occurrences in Thailand in 2014 and used a shallow landslide instability prediction model (SLIP) to foretell the risks of landslides based on rainfall triggering factor in 2018 [24,25]. Almost all research predicts the likelihood of landslides for an area at the provincial, district, or sub-district level. However, the landslide prediction of smaller areas, at the village level or smaller, have not been largely presented. Therefore, the smart GIS web application of landslide-prediction risk is an important challenge for this country.

The prediction of landslides is the one of the most challenging topics because of the complexity of the relationships of various dynamic and uncertain factors and the physical gaining data processes. When the relatively small or limited number of landslide data are available that will lead to an extreme scenario of the landslide prediction problem. The prediction method will be formulated to capture the significant features from the training model process. This research exploited state-of-the-art machine learning approaches for building landslide prediction applications, given by limited historical landslides and landslide factors. The innovations of this research can be highlighted as the following points: Firstly, only five landslide factors from three historical landslides were provided for model learning, categorized into 2 groups: statistical factor and dynamic factor. Secondly, various types and formats of data; i.e., aerial digital images, Google satellite, and site survey photographs, were combined by using QGIS software tool to form geospatial landslide information. The learning weight of the full-connected network will be determined by the value from the previous associated training model. Next, the model-learning approaches characterize the nonlinear long-term scenarios to describe the multi-scale temporal and spatial relationships between landslide occurrence and various influencing factors [26]. To the best of our knowledge, based on literature reviews, Bidirectional-LSTM (Bi-LSTM) has rarely been applied for the susceptible landslide-prediction problem yet. The prediction model automatically corresponds to dynamic factors i.e., rainfall. Finally, a web application of dynamic online landslide susceptible risk was developed through interfacing dynamic rainfall from API data, the trained model, and geometric visualization via website.

This paper is organized as follows: Section 2 presents the detail of the landslide-risk web GIS methodology. Next, Section 3 evaluates and elucidates the performance of the proposed landslide-risk web GIS method. Finally, Section 4 summarizes the proposed web application and future research prospects.

## 2. Methodology

### 2.1. Study Area

**Figure 1:**
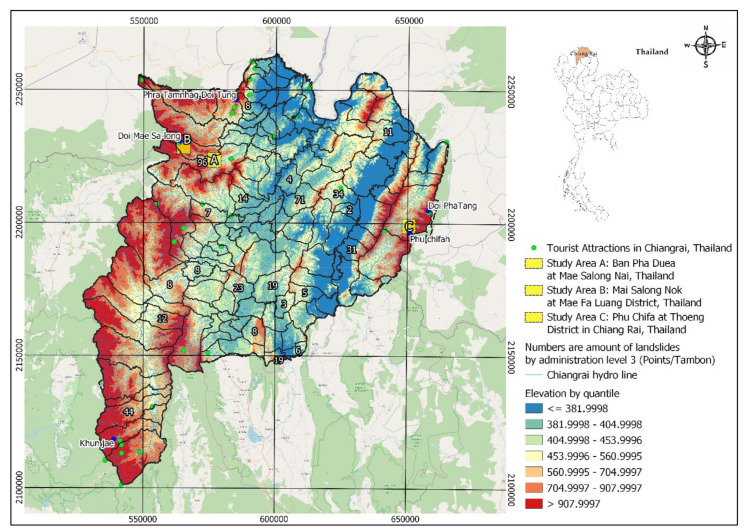
Digital map of historical landslide locations in the study area.

Chiang Rai Province in Thailand is the one of the country’s most famous tourist attractions. Chiang Rai is Thailand’s northernmost province and is mostly a high mountainous and hilly region. The average elevation of the province is 580 m (1903 ft), where the highway stretches through seven large mountains and the Kok river flows along Chiang Rai’s north side. Chiang Rai has a tropical wet and dry climate with an average daily maximum of 34.9 °C (94.8 °F) [27]. The rainy season runs from late April through October. The studies are three historical landslide locations which are Ban Pha Duea (A) at Mae Salong Nai, Mai Salong Nok (B) in Mae Fa Luang District, and Phu Chifa (C) at Thoeng District, Chiang Rai, Thailand as presented in Figure 1. These areas have frequently occurring landslides in tourist and densely populated areas.

**Figure 2:**
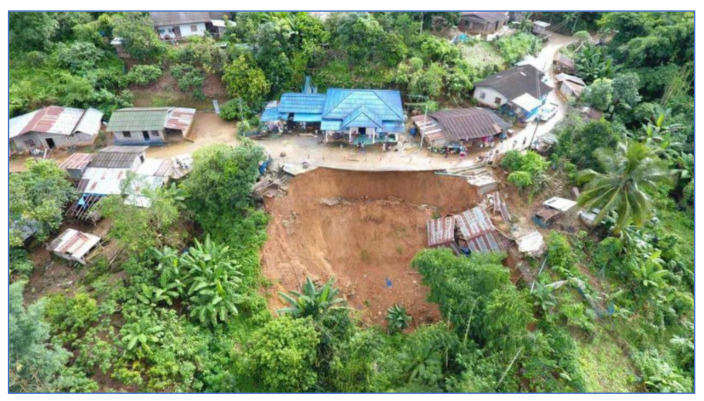
Landslide on 4 September 2017 at Pha Dua Village Community (Study Area A). Ref. https://www.thairath.co.th/news/local/north/1062644 (accessed on 30 May 2021).

Pha Dua Village Community (Study Area A), which is located in Mae Salong Nai Subdistrict Mae Fah Luang District and near the area of Tambon Pa Tung, Mae Chan District. Study Area A covers an area of 25 square kilometers. Study Area A has traces of landslides in several places as shown in Figure 2.

In the recent past, in 2017, a landslide occurred in the area of community in Village No. 6, Ban Pha Dua. Whole houses collapsed and were damaged by the movement of the soil in a vertical distance of over 40 m, sliding down to a depth of more than 30–40 m. The proportion of land cover types of Study Area A are 6.26%, 3.50%, 60.08%, 23.92%, and 6.24% for building, road, trees, glass or shrub, and bare soil, respectively. Rock type is Dc. The elevation of Study Area A is in the range of 630–550. The slope level of Area A is in the range of 0.04–53.5.

**Figure 3:**
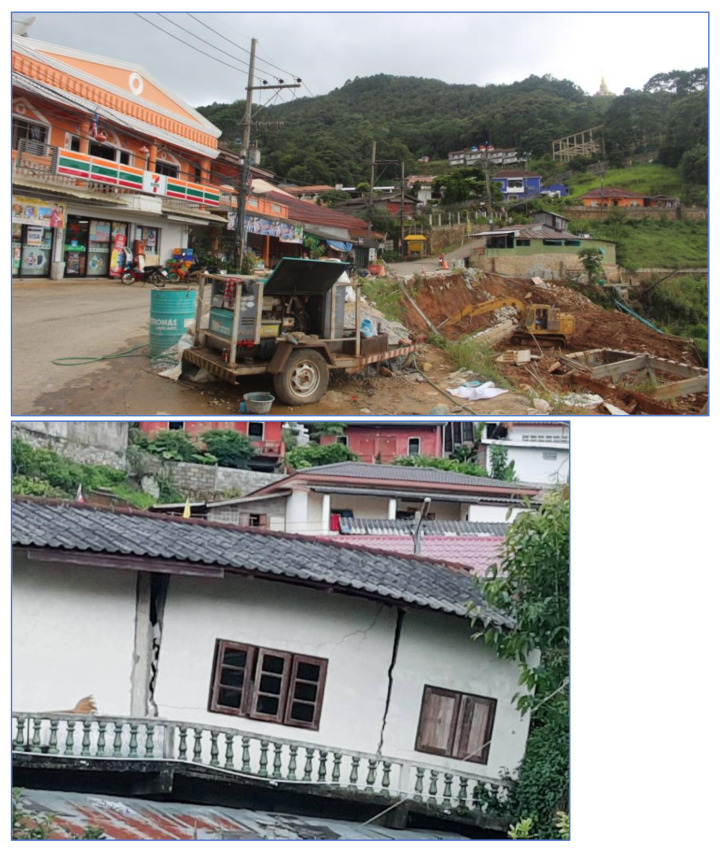
Landslide on 21 August 2018 at Mai Salong Nok (Study Area B). Ref. https://mgronline.com/local/detail/9610000083606 (accessed on 30 May 2021).

Study Area B refers to a landslide that occurred 21 August 2018, where soil in the community area sank more than 25 cm deep and stretched over 80 m as shown in Figure 3. The proportion of land cover types of study area B are 20.58%, 7.66%, 47.78%, 19.02%, and 4.96% for building, road, trees, glass or shrub, and bare soil, respectively. Rock type is Cb. The elevation of Study Area B is in the range of 1080–1100 m. The slope level of Area A is in the range of 0.03–56.9.

**Figure 4:**
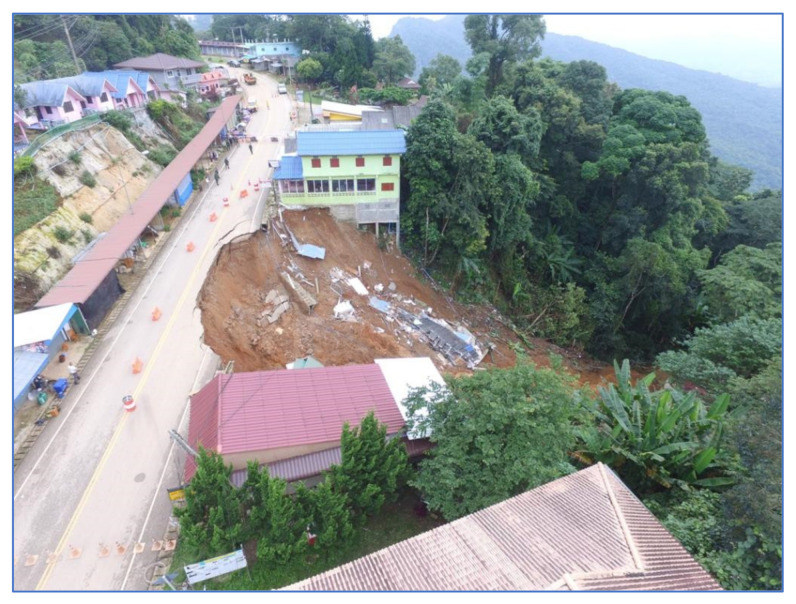
Landslide on 31 July 2018 at Phu Chifa, (Study Area C). Ref. https://mgronline.com/local/detail/9610000075917 (accessed on 30 May 2021).

Study Area C is a landslide that occurred on 31 July 2018, where buildings collapsed into a canyon about 50 m deep as shown in Figure 4. The proportion of land cover types of Study Area C are 12.51%, 10.24%, 25.56%, 33.24%, and 18.44% for building, road, trees, glass or shrub, and bare soil, respectively. Rock type is Dc. The elevation of Study Area C is in the range of 1260–1340. The slope level of Area A is in the range of 0.01–68.6.

### 2.2. Instability Landslide Factors

**Figure 5:**
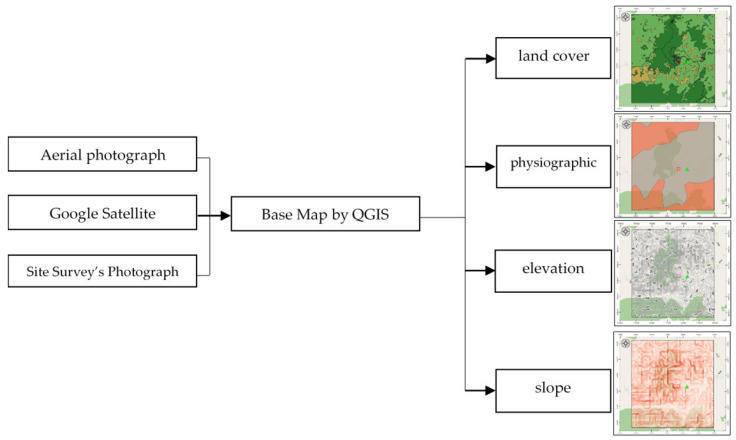
Static instability-landslide factors by QGIS.

Four static factors are considered for landslide-risk prediction, namely, land cover, Physiographic (soil properties), elevation and slope, and a single dynamic factor i.e., precipitation [28]. Static data acquisition is achieved using QGIS software to interpolate contour, elevation, slope degree, and land cover from the Google satellite images and aerial and site survey photographs, while physiographic and rock type are on-site surveyed by experts. Figure 5 presents static instability-landslide factors by QGIS.

**Figure 6:**
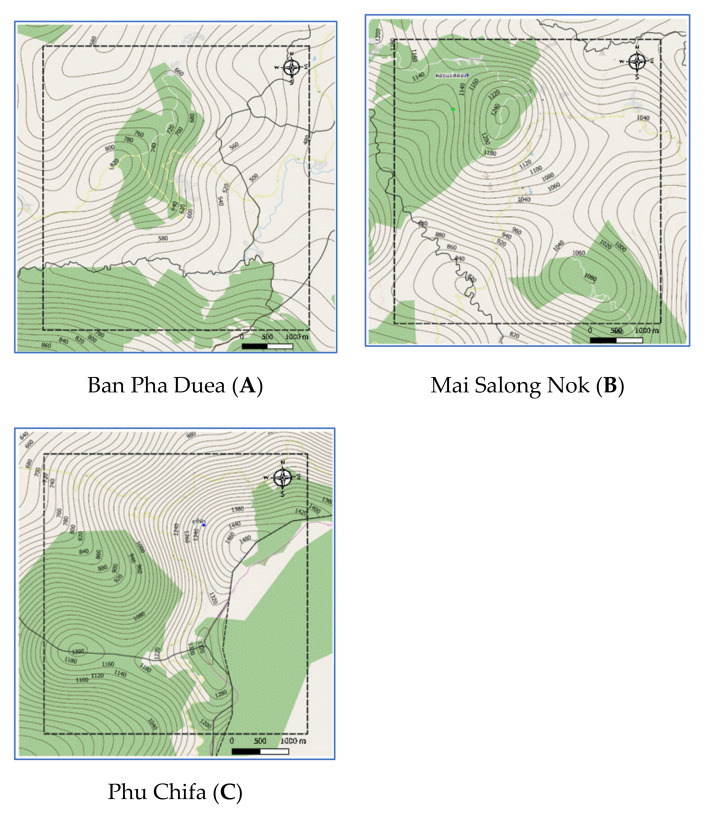
Study area of A, B, and C by QGIS.

Study Areas: A, B, and C have latitude-longitude at the center of each area as: (20.169946, 99.7401521), (20.1537033, 99.62243674), and (19.8446844, 100.4371284), respectively. Each study area covers an area of 25 square kilometers. Geographical data of the three landslide sites was obtained from 3 sources: aerial photographs, Google satellite, and site surveys. Aerial photographs are primary geographic data obtained from relevant government agencies such as Department of Mineral Resources, Ministry of Natural Resources and Environment, Thailand, Royal Thai Survey Department, and Bureau of Research, Development and Hydrology in Department of Water Resources. Google Satellite refers to the digital aerial photographs taken by selecting the satellite view of Google Maps web application. Geographic information is used for determining the position of the landslide locations from the detailed satellite data of the study areas i.e., A, B, and C as in Figure 6. The digital evaluation maps (DEM) were generated by using QGIS according to the following steps:

1. Determine the center of the study area based on an EPSG coordinate system.
2. Generate based map by using QGIS tools.
3. Segment the area into grid cells where a grid size is 0.25 km^2^.

#### 2.2.1. Land Cover Geographic Information

Land cover was categorized into 6 types given by Google satellite, which are bare soil, building, road, glass or shrub, and trees. The land-cover maps were captured in 2019. The 6 types of land cover were classified according to the land-cover classes of the Land Development Department, Thailand. Aerial photography and Google Satellite images were transformed into digitalized map. Land cover information was generated by the following processes:

1. Annotate each type of land cover by using polygon tools throughout the map.
2. Generate land cover dataset by exporting land cover layer into a Shape file.

**Figure 7:**
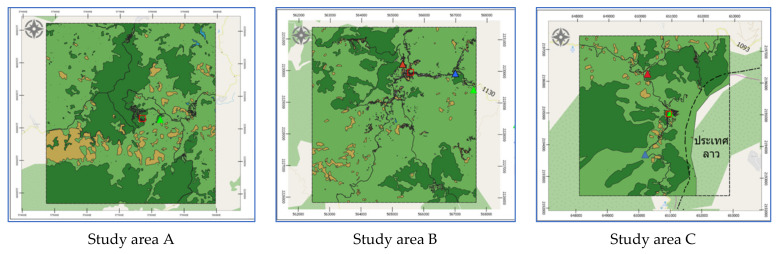
Land cover map in 2019 of study areas: A, B, and C by QGIS.

Examples of land cover map are illustrated in Figure 7 where red square symbol denotes a historical landslide location, dash lines represent a study area, brown areas mean bare soil, green areas represent trees, a light green area is grass or shrub, light gray areas indicate buildings, dark gray areas mean roads, and a blue area means water.

#### 2.2.2. Physiographic (Soil Types) Geographic Information

**Figure 8:**
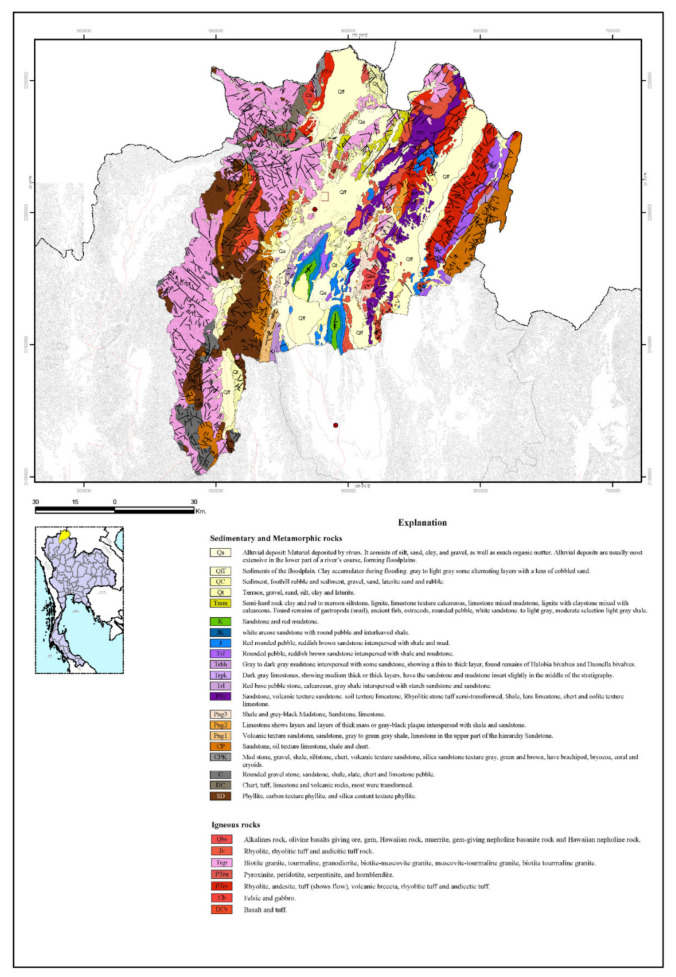
Chiang Rai Geological Map from the Department of Mineral Resources.

Based on aerial photographs from Department of Mineral Resources, Ministry of Natural Resources and Environment, Thailand, Chiang Rai’s physiographic consists of five major classes. Studied areas contain two physiographical classes i.e., the first class is sedimentary or metamorphic rock, and granite in the second class. Aerial photographs were transformed into image digitization via QGIS, as illustrated in Figure 8.

#### 2.2.3. Elevation Geographic Information

**Figure 9:**
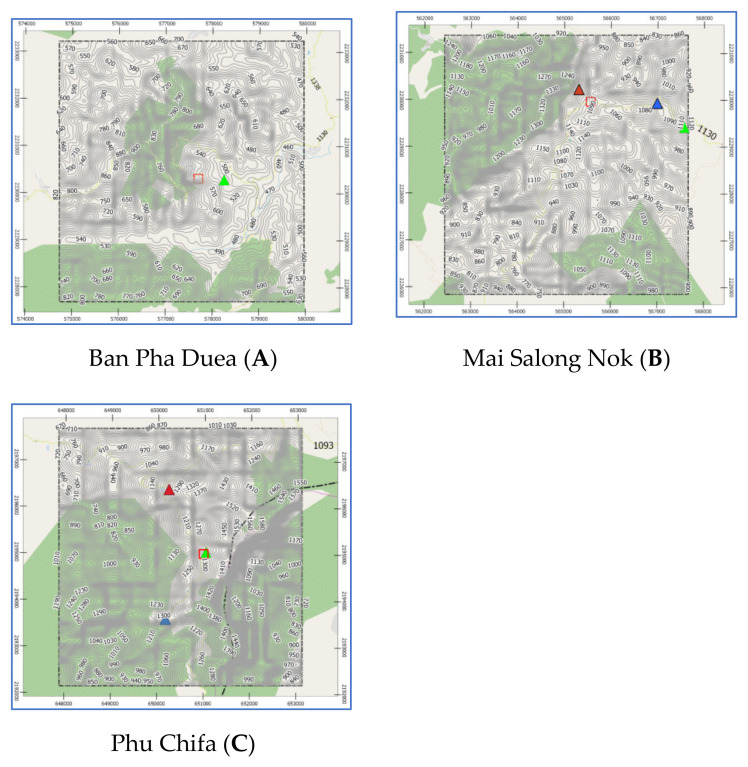
Elevation maps of study areas: A, B, and C by QGIS.

Aerial photographs and DEM from Department of Mineral Resources, Ministry of Natural Resources and Environment and Royal Thai Survey Department were transformed via QGIS by the following steps to generate contouring and elevation map (as presented in Figure 9):

1. Create Raster layer by using DEM. Raster data is like any image that depicts various properties of objects in the real world.
2. Generate contouring and elevation by using Vector > Geometry tools > Centroids, set interval between contour line, and fetching elevation values from Raster layer via Sample Raster Values tools.

#### 2.2.4. Slope Geographic Information

**Figure 10:**
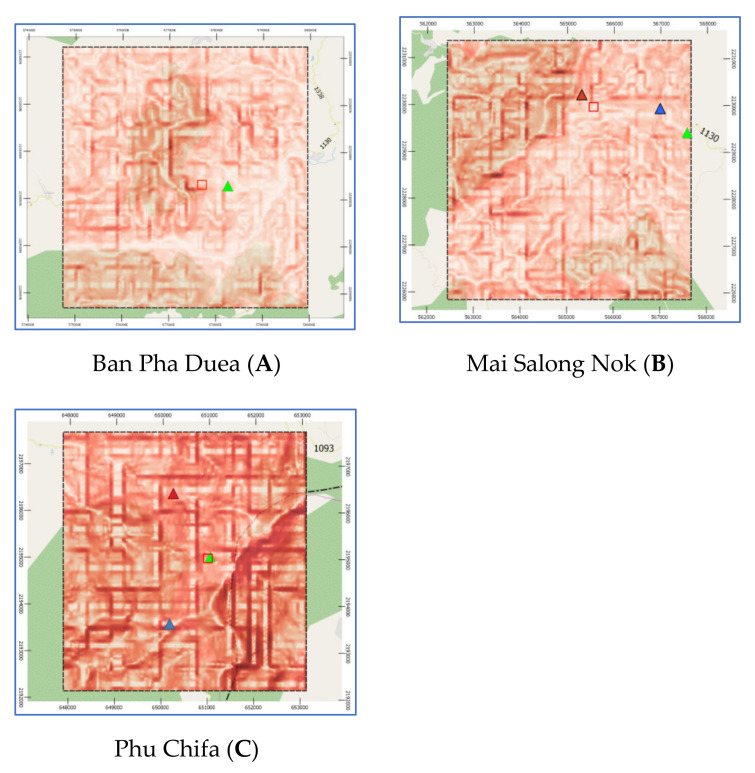
Slope maps of study areas: A, B, and C by QGIS.

Slope is the angle of disposition to the horizontal. Slope was obtained using primary data from aerial photographs and DEM from Department of Mineral Resources, Ministry of Natural Resources and Environment and Royal Thai Survey Department. Slope maps were rendered by QGIS via Slope tool to illustrate the steepness of areas, as shown in Figure 10. The steepness (degree) of the slope is expressed by its saturation—steeper slopes are darker colors.

In the Figure 7, the symbols 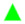, 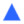, 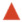, and 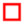 denote Subdistrict Administrative Organization, school, temple, and landslide area. Slope level 
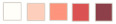
 symbols are 0.03, 15, 30, 45, and 60, respectively.

#### 2.2.5. Dynamic Rainfall Factor

**Figure 11:**
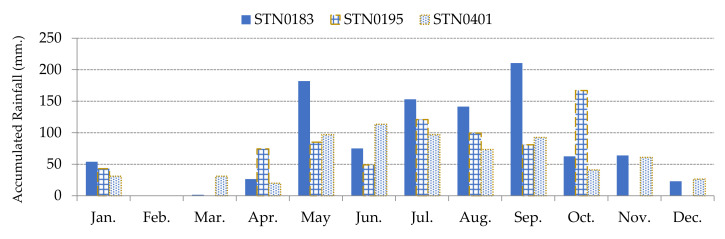
The 4-day cumulative rainfall of 3 rainfall monitoring stations in 2017 of Study Area A and B.

**Figure 12:**
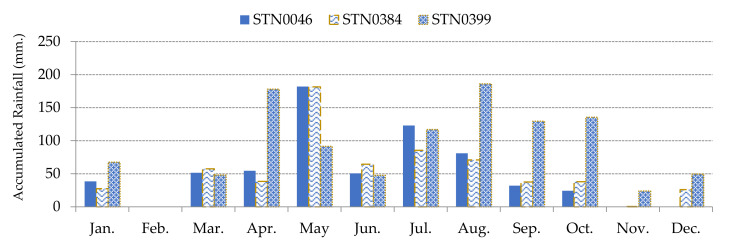
The 4-day cumulative rainfall of 3 rainfall monitoring stations in 2017 of Study Area C.

The cumulated rainfall and the duration of the rainfall events can trigger landslides but in the case of less severe rainfall events, moisture content plays an important role in the occurrence of landslides. Heavy rain breaks the bond between soil particles. This causes the particle disintegration. Continuous flow of runoff water along land covers leads to slides. Due to soil absorbing water, the unit area and total weight increases, and also in pore pressure. Finally, the water percolates in joints and cracks and triggers landslides [29,30]. Daily rainfall data was collected from the Bureau of Research, Development and Hydrology in Department of Water Resources, Ministry of Natural Resources and Environment, Thailand. Rainfall of each study area is based on the three closest rainfall monitoring stations. The 4-day cumulative rainfall was calculated for the dynamic in rainfall as plotted in Figure 11 and Figure 12. The 4-day accumulated rainfall is the summation of the daily rainfall and the previous 3 days of precipitation. Studied areas A and B are close to each other. Hence, both areas use the same rainfall monitoring stations, which are STN0183, STN0195, and STN0401. However, studied area C uses the rainfall monitoring stations: STN0046, STN0384, and STN0399. Effect of rainfall on landslides is interpolated by relating distance from each rainfall monitoring station of the individual area to each grid cell. Thus, the effects of drainages and surface water in the areas were interpolated by computing a ratio of rainfall per the distance between the centroid of each grid cell to each rainfall monitoring station.

### 2.3. Landslide-Risk Prediction Models

The five model-based methods were investigated for forecasting landslide-risk prediction: logistic regression (LR), artificial neural network (ANN), gated recurrent units (GRU), LSTM, and bidirectional LSTM (Bi-LSTM).

#### 2.3.1. Logistic Regression

Logistic regression (LR) is an extension of the linear regression for classification binary problems. LR is a statistical method similar to linear regression, since LR finds an equation that predicts an outcome for a binary variable [31]. LR does not strictly require continuous data. To predict group membership, LR uses the log odds ratio rather than probabilities and an iterative maximum likelihood method rather than a least square to fit the final model. This means the researcher has more freedom when using LR and the method may be more appropriate for non-normally distributed data or when the samples have unequal covariance matrices. Logistic regression assumes independence among variables, which is not always met in morphoscopic datasets.

LR can be expressed as the general equation in Equation (1) that can handle any number of numerical and/or categorical variables [32].

(1) $$p=\frac{1}{1+e^{-\left(\beta_{0}+\beta_{1}X_{1}+\beta_{2}X_{2}+\ldots +\beta_{n}X_{n}\right)}}=\frac{1}{1+e^{-\left(\beta_{0}+\sum_{i=1}^{n}\beta_{i}X_{i}\right)}}$$

LR equation produces a logistic curve, that the probability of the outcome (p) is limited between 0 and 1 given by a vector data ($X_{n}$). The constant ($\beta_{0}$) interchanges the curve left and right and the slope ($\beta_{n}$) defines the steepness of the curve. An advantage of logistic regression is that it allows the evaluation of multiple explanatory variables by extension of the basic principles. Logistic regression can also be used to solve problems of classification as argument of the sigmoid function. The corresponding output of the sigmoid function is a number between 0 and 1. The middle value is considered as threshold to establish what belongs to the class 1 and to the class 0.

#### 2.3.2. Artificial Neural Network

**Figure 13:**
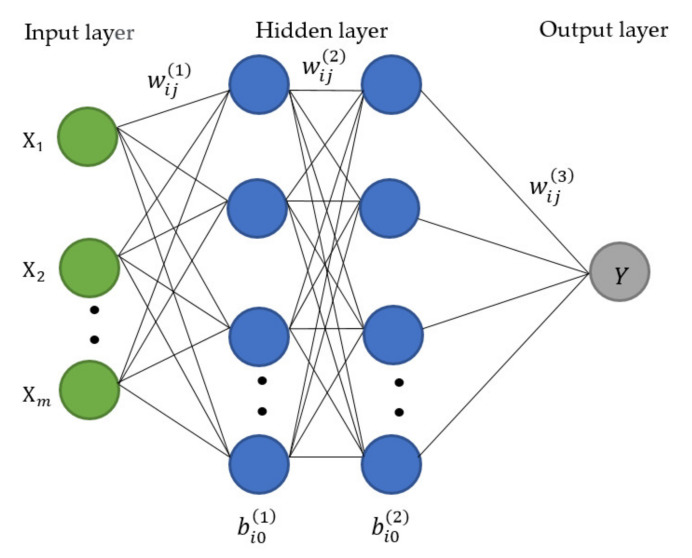
Artificial neural network architecture.

Artificial neural networks (ANNs), usually simply called neural networks (NNs), are computing systems inspired by the biological neural networks of the human brain processes information [33,34,35,36,37]. ANN consists of a first input layer, then hidden layers, and lastly an output layer [38]. In each of the layers in ANN, there are nodes called neurons. A node is a process of a sum of the data along with its coefficient and transfer function through the network. The process is illustrated in Figure 13.

The neurons network of multi hidden layers can expressed in Equation (2).

(2) $$x_{i}^{\left(l+1\right)}=f^{\left(l+1\right)}\left(\overset{j}{\underset{i=1}{\sum }}w_{ij}^{\left(l\right)}x_{j}^{\left(l\right)}+b_{i0}^{\left(l\right)}\right)$$

where $x_{j}^{\left(l\right)}$ denotes the *j*^th^ input at the *l*^th^ layer e.g., *l* = 1, 2, 3, and so on. The term $w_{ij}^{\left(l\right)}$ indicates the weight of the *l*^th^ layer connecting the *j*^th^ node to the *i*^th^ node in the next layer, $b_{i0}^{\left(l\right)}$ is the bias term of the *l*^th^ layer connecting to the *i*^th^ node, and $f^{\left(l\right)}\left(\cdot \right)$ is the activation function of the *l*^th^ layer. The rectified linear units (ReLU) and the Sigmoid function are the two types of the activation functions that are used in this research. The ReLU and sigmoid function can be expressed as illustrated in Equations (3) and (4), respectively.

(3) $$f_{ReLU}\left(x_{j}^{\left(l\right)}\right)=max\left(0,x_{j}^{\left(l\right)}\right)$$

(4) $$f_{sigmoid}\left(x_{j}^{\left(l\right)}\right)=\frac{\exp\left(x_{j}^{\left(l\right)}\right)}{\exp\left(x_{j}^{\left(l\right)}\right)+1}$$

The prediction accuracy of the ANN depends on the number of neurons in the hidden layer; as high numbers of neurons have been shown to enhance the closeness of model outputs with the training data, although this is not always the case. ANN approach is applicable to all kinds of relationships including nonlinear, piecewise, discontinuous, etc.

#### 2.3.3. Gated Recurrent Units

**Figure 14:**
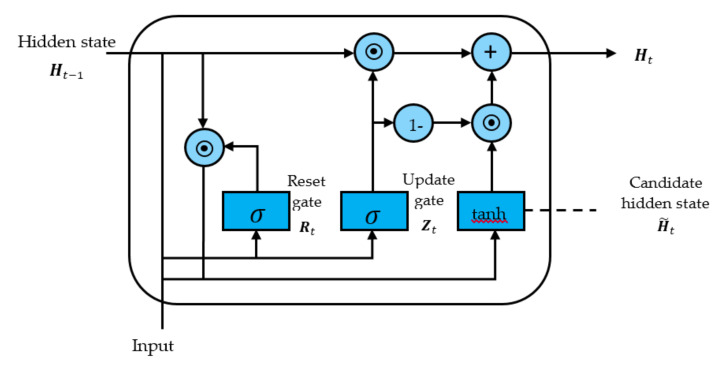
Structure circuit of gated recurrent unit model.

Gated Recurrent Units (GRU) is an improved version of standard recurrent neural network [39,40]. GRU diagram is illustrated in Figure 14.

GRU uses update gate and reset gate to decide [41] which information should be passed to the output. The update gate $z_{t}$ for time step t using the formula in Equation (5):

(5) $$z_{t}=\sigma \left(W_{z}\;\cdot \;\left[h_{t-1},x_{t}\right]\right)$$

when $x_{t}$ is plugged into the network unit, it is multiplied by its own weight $W_{z}$. The same goes for $h_{t-1}$ which holds the information for the previous t−1 units and is multiplied by its own weight. Both results are added together, and a sigmoid activation function is applied to squash the result between 0 and 1.

The gate is used from the model to decide how much of the past information to forget as expressed in Equation (6).

(6) $$r_{t}=\sigma \left(W_{r}\cdot \;\left[h_{t-1},x_{t}\right]\right)$$

where multiply $h_{t-1}$ and $x_{t}$ with their corresponding weights, sum the results and apply the sigmoid function. Current memory content will use the reset gate to store the relevant information from the past. It is calculated as follows in Equation (7):

(7) $${\tilde{h}}_{t}=tanh\left(W\left[r_{t}*h_{t-1},x_{t}\right]\right)$$

Final memory at current time step and information for the current unit is passed down to the network for updating gate. It determines what to collect from the current memory content that is done as follows in Equation (8):

(8) $$h_{t}=\left(1-z_{t}\right)*h_{t-1}+z_{t}*{\tilde{h}}_{t}$$

#### 2.3.4. LSTM

Machine supervised learning techniques have been used to analyze the spatial distribution of landslides by modeling the predictor on a probability of landslide occurrence. The conventional RNN can memorize short-term sequence data but cannot reserve long-term sequence data. Hence, LSTM is a memory module of RNN that preserves and transfers the long depending data through the network. The precipitation factor is regarded as rainfall-induced landslides. A rainfall scenario is a duration of continuous precipitation from the rainfall periods before and after the landslide scene [41,42,43]. The machine will learn the occurrence of landslide and non-landslide data in order to capture the significant landslide pattern. Thus, Bidirectional-LSTM was employed to learn the factor data of forward and backward information. This Bi-LSTM aimed to model the inter-relationship among the factors from sequential observed data [44,45].

**Figure 15:**
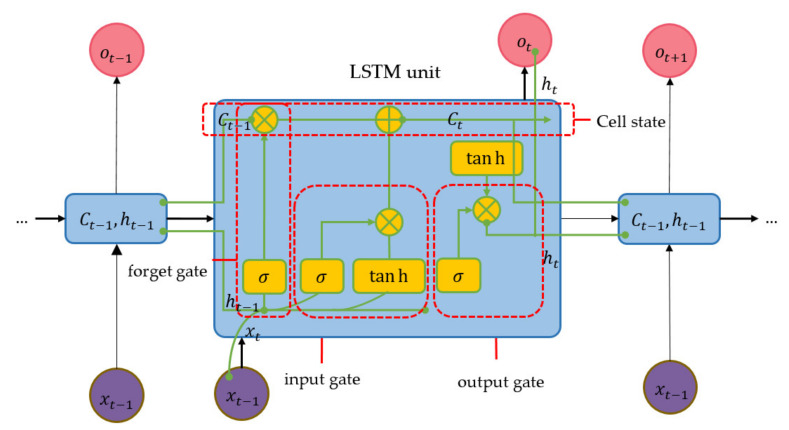
Structure circuit of LSTM model.

LSTM consists of three types of gates that are forget gate ($f_{t}$), input gate ($i_{t}$), and output gate ($o_{t}$). The gates selectively memorized the substantial parameters of the gradient-decrease function. The overview of LSTM is illustrated in Figure 15, composed of two major components i.e., hidden layers (h) and cell states (C), where the t and t−1 terms orderly represent the previous time, and the current time.

Forget gate will decide to reject or reserve information from the cell state (C) that can be expressed as in Equation (9). The output of the forget gate is then input vector ($x_{t}$), which corresponds to the value of the previous hidden layer.

(9) $$f_{t}=\sigma \left(W_{f}\;\cdot \;\left[h_{t-1},x_{t}\right]+b_{f}\right)$$

where σ denotes an activation function i.e., Sigmoid function, W and b are weight matrix and bias vector, respectively, for $f_{t},\;i_{t},\;j_{t},$ and $o_{t}$. Input gate’s components expressed in Equation (10) will compute the information to be updated as a new value ($j_{t}$) by tanh function in Equation (11).

(10) $$i_{t}=\sigma \left(W_{i}\cdot \;\left[h_{t-1},x_{t}\right]+b_{i}\right)$$

(11) $$j_{t}=tanh\left(W_{j}\cdot \;\left[h_{t-1},x_{t}\right]+b_{j}\right)$$

The current cell ($C_{t}$) is obtained by adding the results from Equations (10) and (11), which are multiplied with the multiplying results of the forget gate and the previous cell-state value ($C_{t-1}$). The value of the current cell can be expressed in Equation (12).

(12) $$C_{t}=f_{t}*C_{t-1}+i_{t}^{*}j_{t}$$

The zero or one of the cell states $C_{t-1}$ means ‘discarded information’ and ‘reserved information’, respectively. The value of output gate can be written as in Equation (13). Next, the value of the current hidden layer computes by relying on the cell status with tanh function as expressed in Equation (8).

(13) $$o_{t}=\sigma \left(W_{o}\;\cdot \;\left[h_{t-1},x_{t}\right]+b_{o}\right)$$

(14) $$h_{t}=o_{t}*tanh\left(C_{t}\right)$$

#### 2.3.5. Bi-LSTM

**Figure 16:**
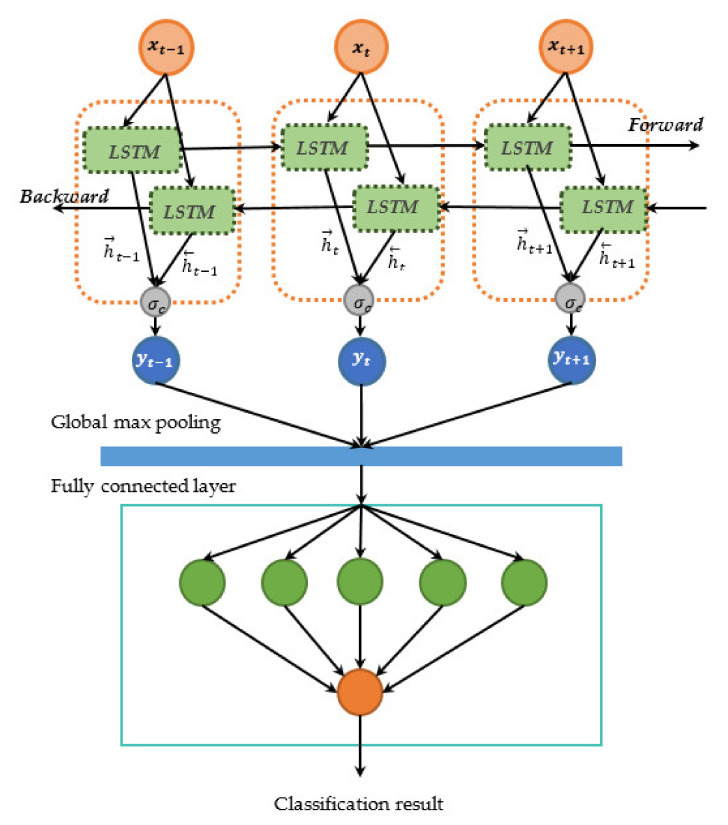
Architecture of Bi- LSTM model.

Bi-LSTM architecture are presented in Figure 16. The Bi-LSTMs are comprised of a forward LSTM (${\vec{h}}_{t}$) and a backward LSTM (${\overset{\leftarrow }{h}}_{t}$) [46]. Both forward and backward layers perform the standard LSTM by using Equations (9)–(14). The forward layer will compute a positive input sequence from time T−n to T−1, while the reverse time sequence from T−1 to T−n for the backward layer. The output of Bi-LSTMs can be expressed as the following Equation (15)

(15) $$y_{t}=\sigma_{c}\left({\vec{h}}_{t},{\overset{\leftarrow }{h}}_{t}\right)$$

where a $\sigma_{c}$ function can be an average function, a concatenating function, a multiplication function, or a summation function for combining the two ${\vec{h}}_{t}$ and ${\overset{\leftarrow }{h}}_{t}$ outputs from the hidden layers. The final Bi-LSTM output is represented as a vector $Y_{T}=\left[y_{T-n},\;\ldots ,\;y_{T-1}\right]$.

### 2.4. Landslide-Risk Model Measurement

Landslide-prediction performance was measured by evaluating confusion metrics into the statistical indexes [17]. The statistical indexes can be expressed in Equations (16)–(18)

(16) $$PPR=\frac{TP}{TP+FP}$$

(17) $$NPR=\frac{TN}{TN+FN}$$

(18) $$TA=\frac{TP+TN}{TP+TN+FP+FN}$$

where PPR, NPR, and PA denote the positive predictive rate of the corrective predicted landslides, negative predictive rate of the corrective predicted non-landslides, and total predictive rate of the corrective predicted results of landslides and non-landslides, respectively. The TP (true positive) and FP (false positive) terms orderly signify the correct and mistaken number of landslide grids. TN and FN terms indicate the non-landslides that are correctly and inaccurately forecasted. The scores of PPR, NPR, and PA are in the range of [0, 1] where the higher value means the better stability of the model.

In addition, Receiver Characteristic Operator (ROC) curve illustrates the performance of a classification model at all classification thresholds based on two parameters i.e., true positive rate and false positive rate. Area Under the ROC Curve (AUC) provides an aggregate measure of performance across all possible classification thresholds. AUC denotes the capability of distinguishing between classes. The Higher the AUC, the better the model is at distinguishing between locations with the landslides and non-landslides.

### 2.5. Proposed Automated Landslide-Risk Web GIS Application

#### 2.5.1. QGIS for Geospatial Information

QGIS is a free and open-source software tool for manipulating geographic information systems (GIS). QGIS stands for Quantum GIS. QGIS functions are map viewing, adding, and editing geospatial data into maps, analysis associated map information, and exporting graphical maps or data files. Additionally, QGIS supports various types of GIS formats; for example, raster, vector, dxf, shapefile, coverage, and also integrates with geodatabases; such as MapInfo, PostGIS, SpatiaLite, Oracle Spatial, and MySQL Spatial databases. QGIS function is versatile as its capabilities can be extended with plugin software packages or written Python or C++ script for performing some specific functions. QGIS have been employed for numerous landslide studies, for example in [47,48,49]. Other GIS software are ArcGIS [50] and SuperMap. However, ArcGIS and SuperMap are license software.

#### 2.5.2. Automated Landslide-Risk Web GIS Application

**Figure 17:**
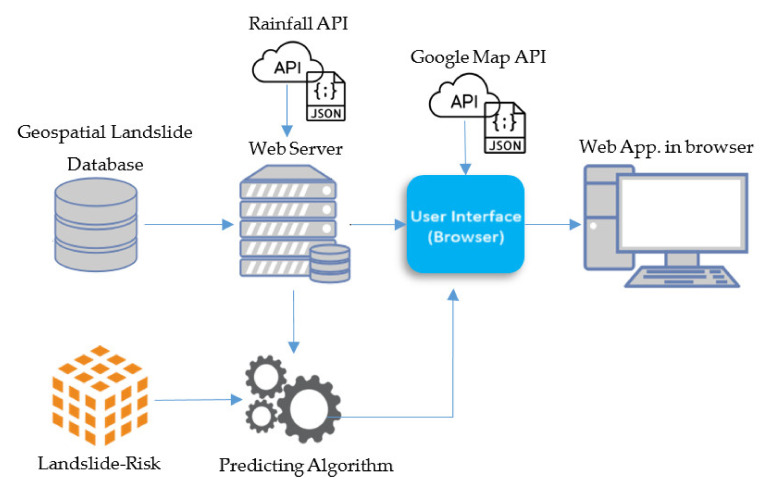
Proposed architecture of landslide-risk automated web application.

An automated web application has been developed to present the degree of landslide risk in study areas A, B, and C. The website will automatically update the risk of landslides every day by fetching the rainfall via an application programming interface (API) from the Bureau of Research, Development and Hydrology in Department of Water Resources. The probability results from the landslide-risk prediction are categorized into one of 5 degrees of landslide risk. The architecture of the website system is shown in Figure 17.

APIs simplifies a complex development of a dynamic GIS website more easily by abstracting more complex code away [51,52]. API cooperates with websites via Hypertext Transfer Protocol (HTTP) methods and JavaScript language [53,54]. According to Figure 17, the working process of the website starts from receiving the rainfall through API as a json file then selecting and fetching the precipitation data of the specified rainfall monitoring stations. The web server is executed in JavaScript language via Node.js tools. Once the daily rainfall has already been obtained, the system calculates the accumulated rainfall for 4 days. The accumulated rainfall along with static landslide-factor data are then passed into the predicting algorithm based on Python language. The predicting algorithm will scale the input data. The scaled accumulated rainfall will be normalized by mean and standard deviation values which are obtained from the training phase. Next, the trained landslide-risk model will be loaded for predicting the landslide-risk probability of the studied areas. Landslide-risk probabilities, latitudes, and longitudes will be sent to front-end section for displaying landslide-risk probabilities of grid cells on Google Maps API. The overview of the proposed landslide-risk prediction algorithm is indicated in the following table Algorithm 1.
**Algorithm 1.** Overview of the proposed landslide-risk prediction algorithm(1) Build the landslide dataset by using QGIS:(1.1)generate grid-based maps of 3 historical landslide occurs of Study areas given by Aerial photographs, Google Satellite, and site survey photographs.(1.2)identify 5 types of land cover from Google Satellite and Aerial photographs and render land cover digital map.(1.3)determine physiographic by using Aerial photographs and site survey photographs.(1.4)compute and render elevation and slope from Aerial photographs and site survey photographs.(1.5)fetch historical rainfall data from Department of Water Resources, Ministry of Natural Resources and Environment, Thailand.(1.6)export all attributes into the CSV files.
(2) Determine the optimal values of batch size, epochs, and the number of nodes in full-connected network of ANN, GRU, LSTM, and Bi-LSTM. (3) Build and train the machine learning model i.e., LR, ANN, GRU, LSTM, and Bi-LSTM by using the optimal values of parameter from Step 2 given by the landslide dataset. The landslide dataset was split into a training dataset (80% of landslide dataset) and a testing dataset (20% of the landslide dataset) (4) Choose the model that delivers the best prediction performance for constructing the web application. (5) Implement and test the automatic landslide-risk prediction web GIS by using JavaScript, Node.js, and MySQL as web programing language, web development tool on server side, and database. (6) Deploy the proposed landslide-risk prediction web GIS on Google Cloud platform

## 3. Experimental Results and Analysis

**Figure 18:**
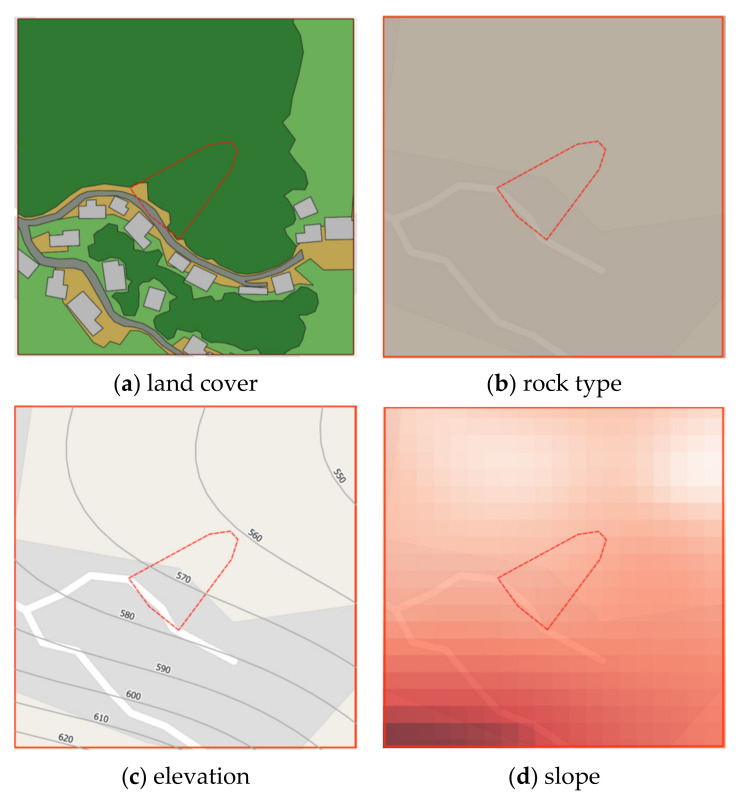
Phu Chifa at Thoeng District, Chiang Rai, Thailand.

**Figure 19:**
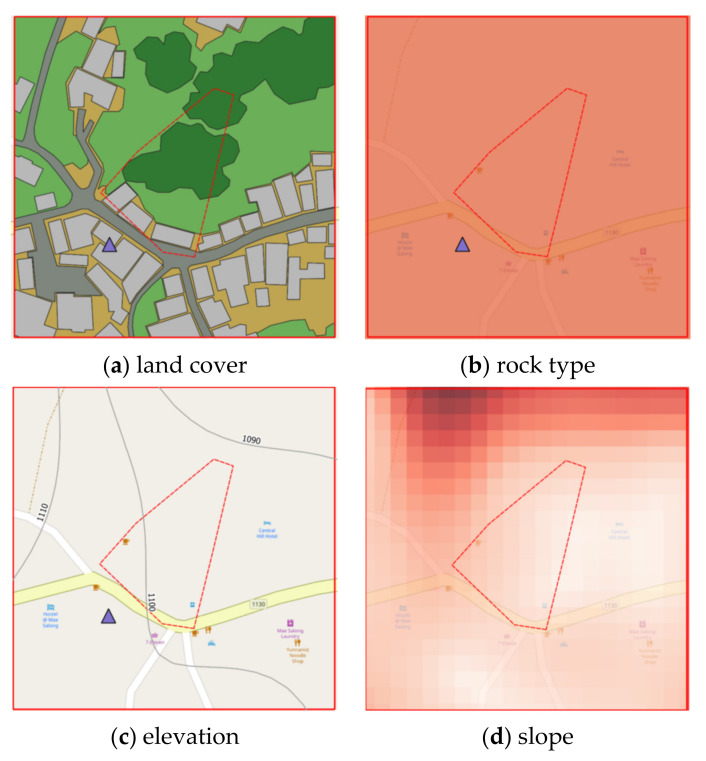
Ban Pha Duea at Mae Salong Nai, Chiang Rai, Thailand.

**Figure 20:**
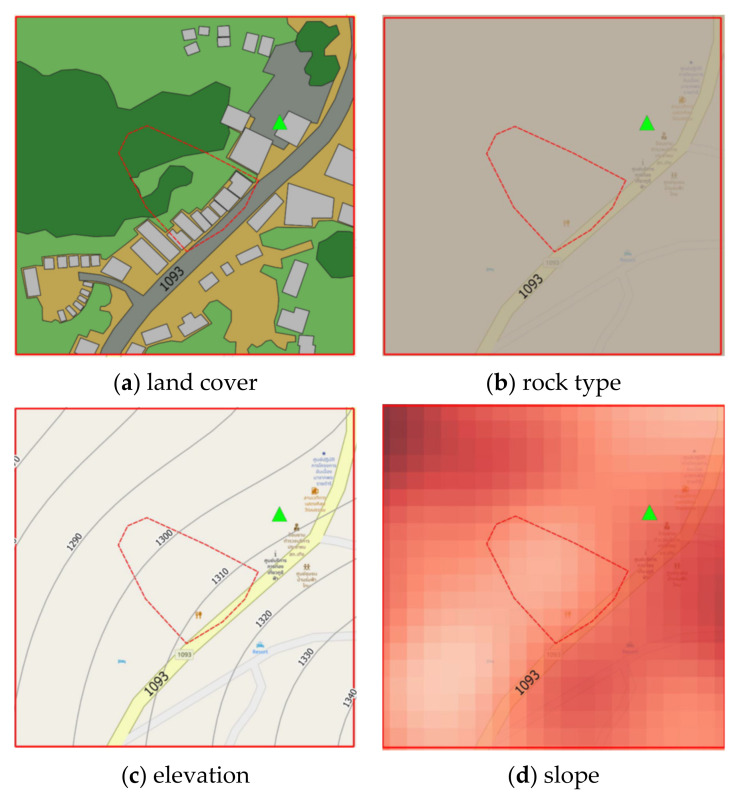
Mai Salong Nok in Mae Fa Luang District, Chiang Rai, Thailand.

The prediction performance of proposed landslide-risk algorithm is investigated in this section, presented in four sections. The first section shows the optimal parameters of LR, ANNs, LSTM, and Bi-LSTM. The second section presents the landslide-predicting performance comparison of the various machine learning methods. An enhanced prediction using two-stage classifiers is proposed in the third section. The fourth section reveals the automation web application for predicting and visualizing landslide risk in Chiang Rai, Thailand. The three historical landslide locations and their landslide factors are presented in Figure 18, Figure 19 and Figure 20.

**Figure 21:**
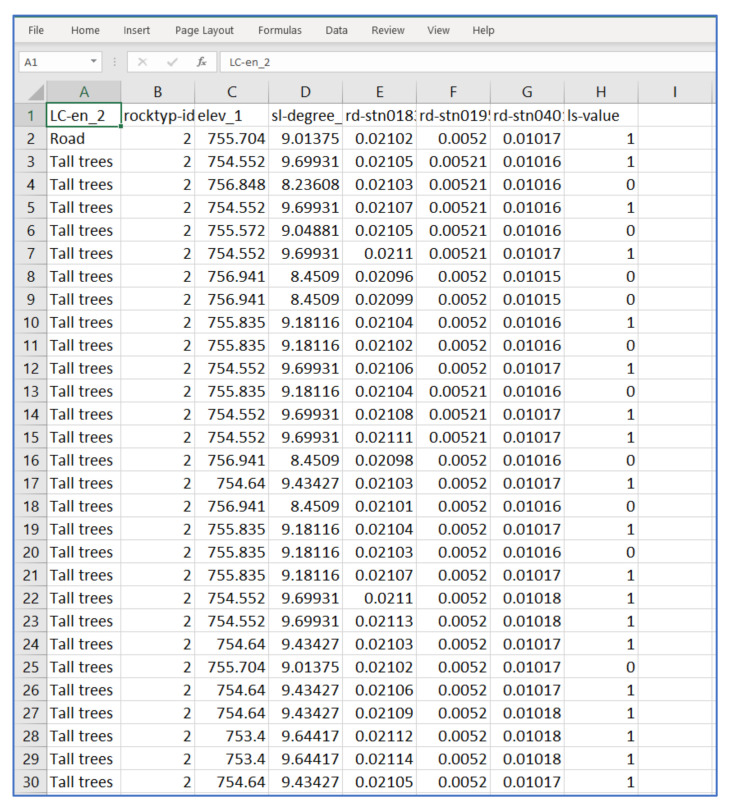
Sample of landslide transaction.

Each site is segmented into 5 m × 5 m grid cells where the grid cell maps are used for training and validating the prediction model. Landslide and non-landslide data were prepared for the supervised learning where landslide transactions were marked as 1 and 0 for non-landslide transactions. Three landslide areas were converted into 1967 grid cells in QGIS software. The landslide dataset was exported from QGIS into a csv format as illustrated in Figure 21, where A–D columns represent static landslide factors, E–G columns contain dynamic factors, and H column is the classes of landslides (1) and non-landslide (0). The numbers of landslide and non-landslide grid cells are 1074 and 893 transactions. The grid data were randomly partitioned by 80% and 20% to be the training and evaluating datasets. The values of 4 static factors and a dynamical rainfall factor: i.e., 3 rain stations for each area; were collected as independent parameters for the machine-learning mode. All input data was normalized to match for reducing the bias learning. The dependent output is likelihood of landslide of each grid that will occur. In determining whether a landslide will occur or not, the outputs were compared to a threshold at 0.5. If probability outputs were less than 0.5, the corresponding grid was marked as non-landslides. Otherwise, the results were marked as landslides.

The experiments were conducted in the following hardware and software environments, hardware environment: AMD Ryzen 9 4900H with Radeon Graphics 3.30 GHz, Nvidia GeForce GTX 1660 Ti, 16.00 GB DDR4, Windows 10, Anaconda v3, Python v3.8.8, TensorFlow-GPU v2.3.0, Keras v2.4.3, and QGIS v3.14.

### 3.1. Determining the Optimal Parameters of Machine Learning Methods

**Figure 22:**
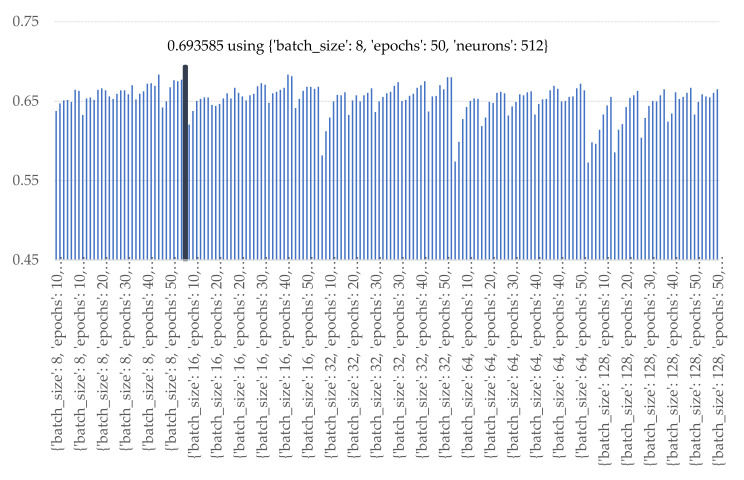
Accuracy of various numbers of batch sizes, epochs, and nodes of ANN1.

Experiments were established to identify the optimal batch size, epochs, and the number of nodes for individual machine learning model i.e., LR, ANN with a single hidden layer (ANN1), ANN with 2 hidden layers (ANN2), ANN with 3 hidden layers (ANN3), GRU, LSTM, Bi-LSTM. The optimal parameterization was conducted according to the following sets [55] i.e., batch size: [8, 16, 32, 64, 128], epochs: [10, 20, 30, 40, 50], and each hidden layer the number of neurons were set as [8, 16, 32, 64, 128, 256, 512]. For all the experiments the parameters of the ANN were configured as the following: an initializing function is a uniform function; activation functions used the ReLU function in hidden layer and the sigmoid function for the output layer. The optimal parametrial results of the machine learning methods are illustrated below. The best landslide-prediction performance of ANN methods was obtained when the number of batch size, epochs, and neurons in the hidden layer were 8, 50 iterations, and 512 nodes as illustrated in Figure 22.

**Figure 23:**
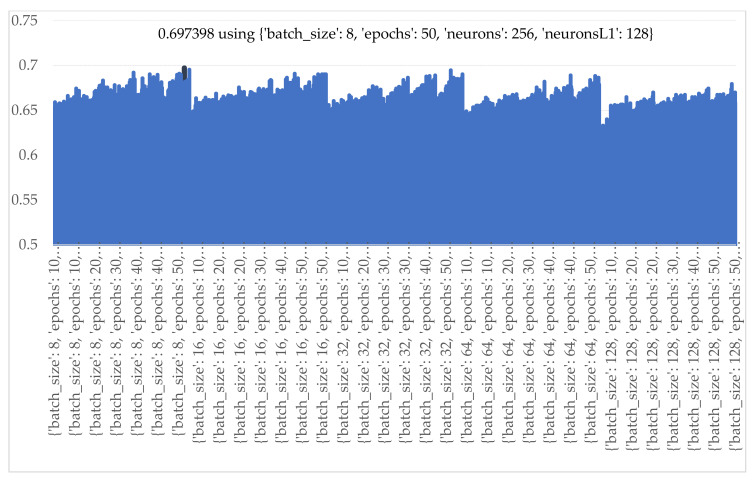
Accuracy of various numbers of batch sizes, epochs, and nodes of ANN2.

The results of ANN with 2nd hidden layers are shown in Figure 23. The best predicting performance was obtained when the number of batch sizes, epochs, and nodes in the 1st hidden layer and the 2nd hidden layer were 8, 50 iterations, 256 nodes, and 128 nodes, respectively.

**Figure 24:**
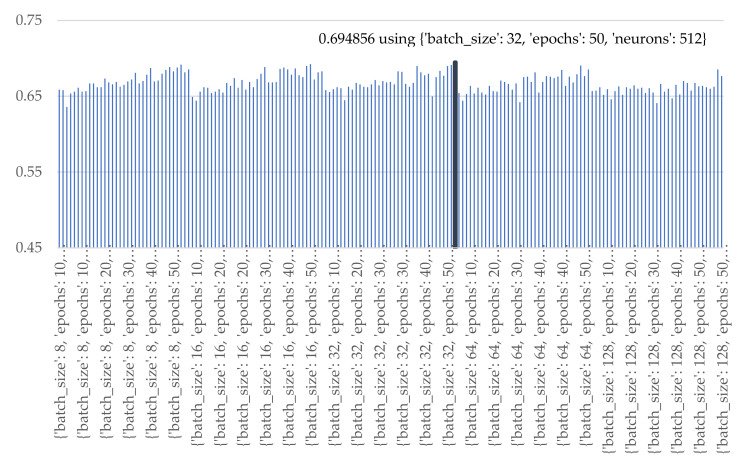
Accuracy of various numbers of batch sizes, epochs, and nodes of the 3rd hidden layer ANN3.

To determine the number of nodes in the 3rd hidden layer, the nodes in the 1st and 2nd layers were orderly set at 256 nodes and 128 nodes according to the previous results. The best landslide-prediction performance of ANN methods was obtained when the number of batch size, epochs, and neurons in the hidden layer were 32, 50 iterations, and 512 nodes as illustrated in Figure 24.

**Figure 25:**
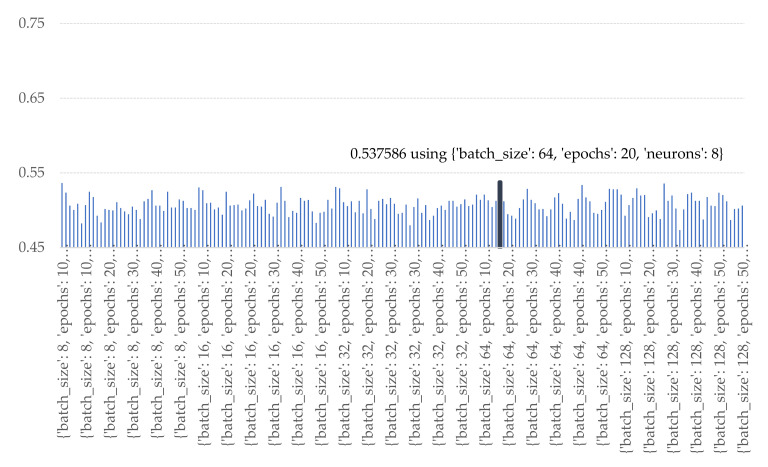
Accuracy of various numbers of batch sizes, epochs, and nodes of GRU.

As per the results in Figure 25, the GRU architecture was configured by batch size at 64, 20 epochs, and 8 nodes for GRU units and full-connected network.

**Figure 26:**
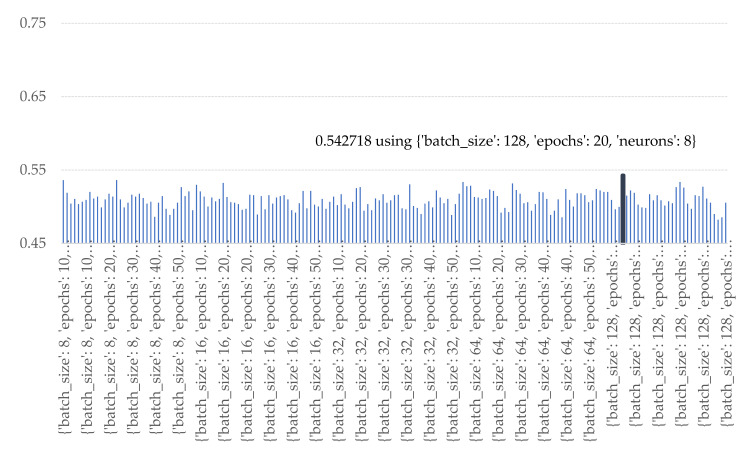
Accuracy of various numbers of batch sizes, epochs, and nodes of LSTM.

**Figure 27:**
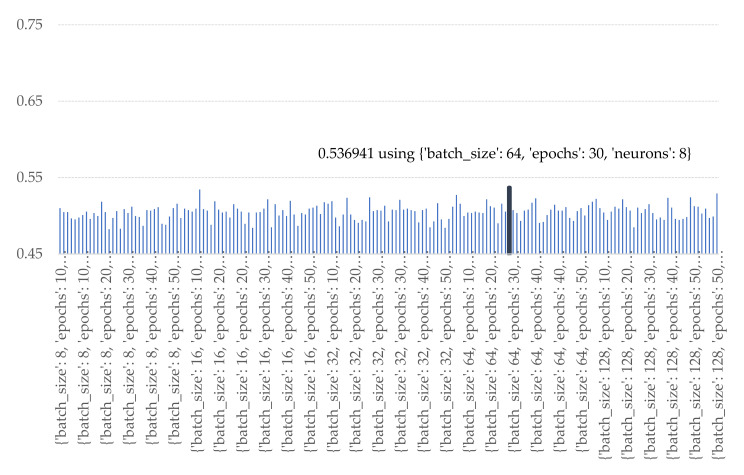
Accuracy of various numbers of batch sizes, epochs, and nodes of Bi-LSTM.

According to the plots in Figure 26 and Figure 27, both LSTM and Bi-LSTM obtained the best prediction performance when the number of nodes was at 8 nodes for both features extracting section and full-connected network. The number of batch size and epochs were 128 and 20 iteration for LSTM method and 64 and 30 iterations for Bi-LSTM method.

**Figure 28:**
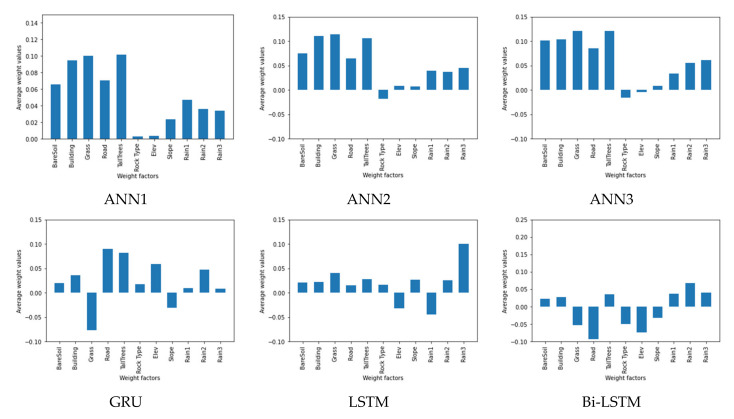
Average weight values of landslide factors of studied machine learning models.

The weight values of the trained models from each method illustrate the importance of landslide-risk factors in causing landslide occurrence. The weight scores of each method are presented in Figure 28. All models showed that rainfall was a significant risk factor of landslides. Moreover, in all models, bare soil, building, and tall trees will affect for landslides as well.

### 3.2. Landslide-Predicting Performance Comparison between Studied Machine Learning Methods

**Figure 29:**
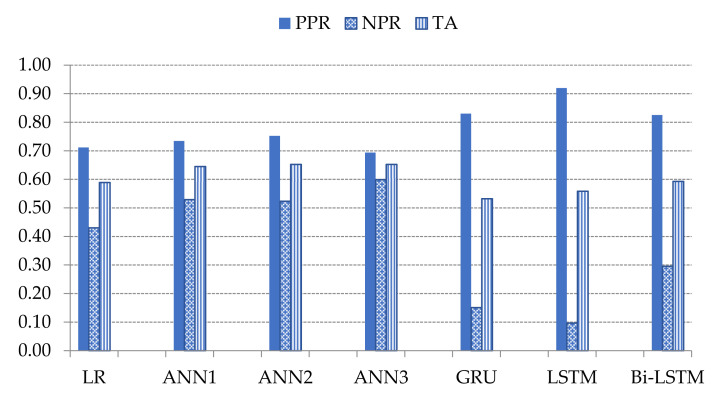
Comparison the values of PPR, NRP, and TA of studied machine learning models.

Based on the experimental results of various parameterization in Section 3.1, the landslide-prediction performance of the trained methods are compared to one another i.e., LR, ANNs, GPU, LSTM, Bi-LSTM. The results are illustrated in Figure 29.

**Figure 30:**
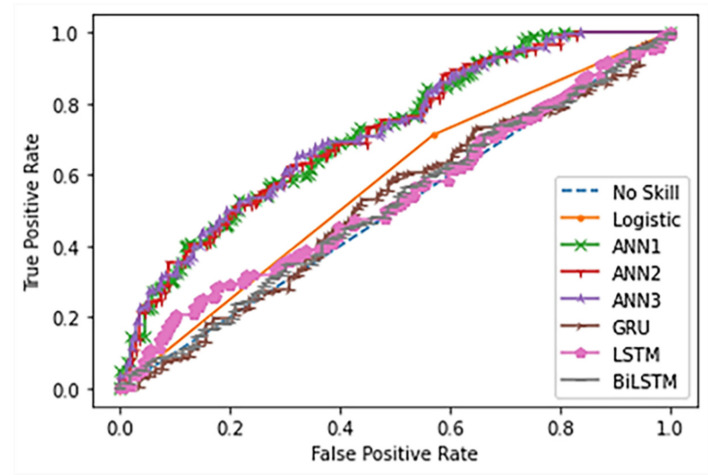
ROC Comparison of landslide-risk prediction among studied machine learning models.

**Figure 31:**
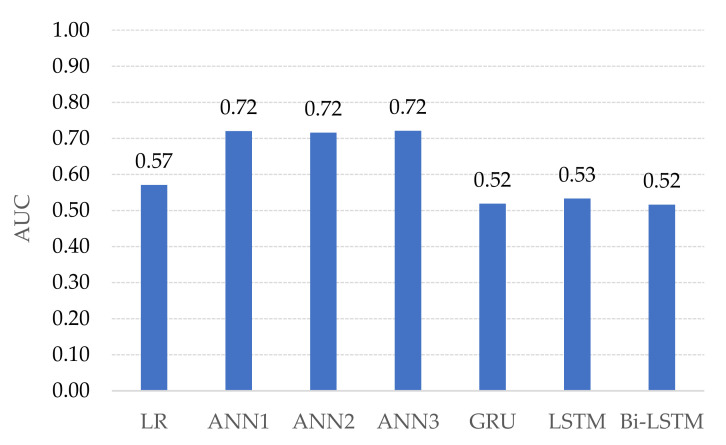
AUC of studied machine learning models for landslide-risk prediction.

GRU, LSTM, and Bi-LSTM yield the best three of PPR at 0.83, 0.92, and 0.83, respectively. The PPR (which can be called a sensitive test) correctly identifies landslides from the all-landslide cases. On the other hand, the best three NPR are the ANNs with triple, double, and single hidden layers at 0.60, 0.52, and 0.53, respectively. The NPR represents the correct identification of non-landslide from all non-landslides. The ANN3 model yields the highest TA score at 0.65 which is 0.12 score higher than GRU, 0.09 score over LSTM and 0.06 score over Bi-LSTM. Figure 30 and Figure 31 present the probability curve with various threshold values and AUC scores. The thresholds of the plots have AUC scores more than 0.5 that can be interpreted as all trained models having a high chance to classify the landslides from non-landslides. The ROC curves of the ANN models are closer to the top-left corner which illustrate better prediction performance and obtain the largest AUC areas among the comparison methods.

### 3.3. Enhanced Prediction: Two-Stage Classifiers

**Figure 32:**
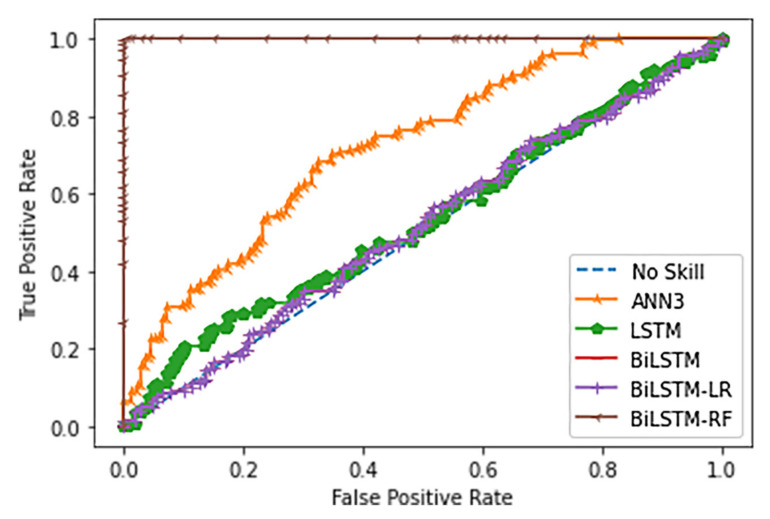
ROC Comparison of 2-stage classifiers with ANN3, LSTM, and Bi-LSTM.

The class of LSTM approaches has superior performance in terms of the positive prediction on many landslide scenarios. However, false alarms have also occurred. Thus, two-stage classifiers have been investigated to improve the performance of LSTM and Bi-LSTM methods given by the limited landslide dataset. Two-stage classifiers are comprised of two sequential steps. The first step is LSTM or Bi-LSTM classification, which aims to estimate the landslide probability of individual grid cells. The next step involves a second classifier connected to the output of the LSTM or Bi-LSTM. The second classifier aims to correct the error resulting from the LSTM or Bi-LSTM and then find the optimal determination of the predicted probabilities from LSTM or Bi-LSTM in order to make a final decision as to whether there is landslide or non-landslide. LR and Random Forest (RF) models have been used for the second classifier step. The output of the first classifier is the independent input of LR and RF model where the actual landslides and non-landslides information are passed to the models as the dependent output. The ROC results of two-stage classifiers (Bi-LSTM-LR and Bi-LSTM-RF) are illustrated in Figure 32.

### 3.4. Landslide-Risk Prediction Model Accuracy

The importance of landslide-risk factors will cause landslide occurrence that are represented by the weight scores as presented in Figure 28. In general, a positive weighting value means that the factor is directly related to the occurrence of landslides or, on the contrary, when it is negative. The site of all three landslides occurred on the middle-elevation area, while at high altitudes there was no landslide. This is because the high-altitude areas are mountains that are covered with rock or forests. This leads to topsoil binding that is stronger than the lower-elevation areas that are residential communities. As a result, elevation does not directly correlate with landslides, which is shown in negative values. In this research, the rock type was comprised of Chert and Quartz where the landslide sites in Study Area: A and C are Chert, while Area B is Quartz. These will cause the weight values of the rock types as both positive and negative values. The results from ANN models show in a consistent direction that land cover is a significant factor for landslides, followed by the rainfall. Factors affecting the formation of landslides of Bi-LSTM model are bare soil, building, tall trees, and rainfall that correspond to the actual environment.

LR is the simplest method for constructing a model and has low computational complexity among all other methods. However, LR is suited for linear relationship between the dependent variable and the independent variables. It is challenging for LR to deal with complex problems or non-linearity in real-world scenarios, such as landslide-prediction problems. The learning network-based models are ANN, GRU, LSTM, Bi-LSTM, and Bi-LSTM-RF. These learning network-based approaches consist of two components which are made up of a learning network and a result. For the first component, determining the optimal learning network architecture and parameters of all models for the problem is critical as it affects the model performance [56]. The network is capable of learning linearity or non-linearity data. At this stage, this dominates the computational complexity of the methods which are generally time consuming compared to LR with the same input dataset. While GRU, LSTM, and Bi-LSTM models are the special cases of the learning network-based approaches, they have memory logics for previous significant data in the learning network model. Therefore, these models will be appropriate for learning a long time-series data. Secondly, in the result decisioning stage, the output can be binary classes, discrete or continuous outcomes which are based on the selected method.

AUC scores in Figure 31 of the landslide-prediction performance show that ANN models yield the highest performance. Due to the occurrence of landslides from the study area surveyed in the past 3 years, only 1 landslide occurrence was found in each area. Therefore, this causes the landslide dataset to have a small amount of transaction. The ANN methods are learning to extract landslide patterns from all datasets, while GRU, LSTM, and Bi-LSTM methods select some data and remove some during learning processes based on the reset or forget gates in the model mechanisms i.e., $r_{t}=\sigma \left(W_{r}\cdot \;\left[h_{t-1},x_{t}\right]\right)$ and $f_{t}=\sigma \left(W_{f}\;\cdot \;\left[h_{t-1},x_{t}\right]+b_{f}\right)$, respectively. Thus, GRU, LSTM, and Bi-LSTM models, which are suitable for long-time scenario dataset, perform landslide-risk prediction at low accuracy when given by the small size of the landslide dataset. If the dataset has added the cumulative rainfall, monthly or throughout the year, it will result in an imbalance classification of the dataset. This is because there is tremendous non-landslide data in comparison with landslides. In addition, an imbalance classification will lead the machine-learning model to over-fit. Therefore, it can be concluded that in the case of the available landslides, landslide influence data are small or limited.

The enhanced prediction model as shown Figure 32 is called the 2-stage classifier Bi-LSTM-RF, and yields superior performance compared to other classifiers. It is also observed that if the second stage classifier is a linear classifier, the ROC performance does not significantly improve. This is evident in the case of 2-stage classifier Bi-LSTM-LR where the ROC result does not differ from Bi-LSTM. As a result, both ROC lines overlap. Since the RF classifier operates a non-linear classification, the probability values from LSTM or Bi-LSTM have been partitioned into multiple ranges of landslide and non-landslide by decision trees in the RF model. Therefore, the 2-stage classifiers can optimally categorize the nonlinearly overlapping results. On the other hand, the LR performs a linear classification and so divides the landslide probabilities into only two ranges: one for landslide and another one for non-landslide. This leads to similar performance with the former LSTM and Bi-LSTM by using the threshold.

One reason that LR, ANNs, GRU, LSTM, and Bi-LSTM obtained an AUC accuracy below 0.8 is due to the challenging dataset that contains small amount of landslides occurrence data. The percentage of landslide and non-landslide in the dataset are 55% and 45%. This dataset has rendered these learning models difficult to discover the correct pattern of landslides. Based on the three historical landslides acquired from the survey site, this limited the number of landslide data, while non-landslide data can be increased by extending the study area in the individual site. As the amount of non-landslide data increases, the AUC score becomes higher to a certain extent. However, our research aims to predict landslides rather than non-landslides. Therefore, we are unable to rely on increasing the amount of non-landslide data. Secondly, this is due to the limitation of the learning method of the model from the small dataset. For example, LR requires the linear assumption, but landslides are nonlinear phenomena. ANNs, GRU, LSTM, and Bi-LSTM require a large amount of data for training the models. Moreover, different locations may naturally have different threshold levels for landslides. When the decision stage of the learning models has a single threshold for determining the estimated probability of landslide or non-landslide for all locations, this impacts on the prediction performance. Hence, we have proposed the Bi-LSTM-RF model which has multi thresholds from the random forest classifier. As the results, the AUC accuracy of the Bi-LSTM-RF method is improved over LR, ANNS, LSTM, and Bi-LSTM by 0.42, 0.27, 0.46, and 0.47, respectively.

### 3.5. Automated Landslide-Risk Web GIS Application

**Figure 33:**
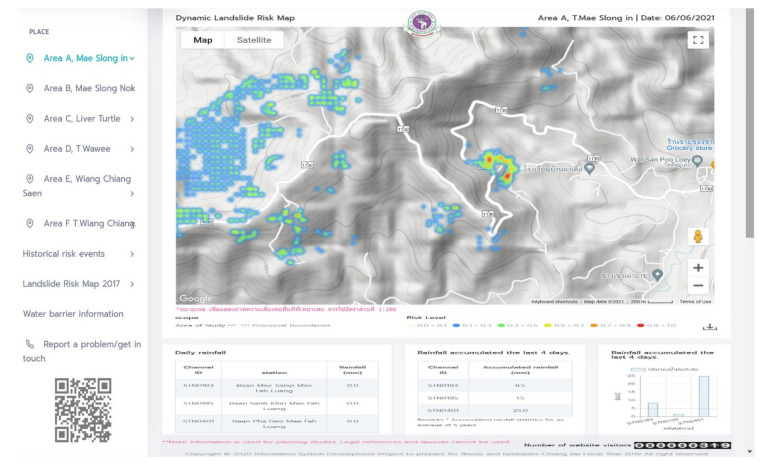
Automated landslide-risk web GIS application.

**Figure 34:**
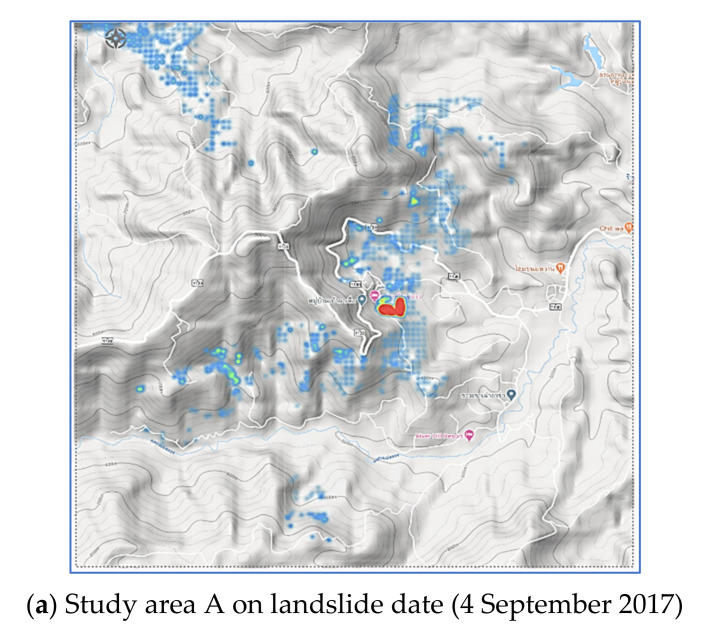

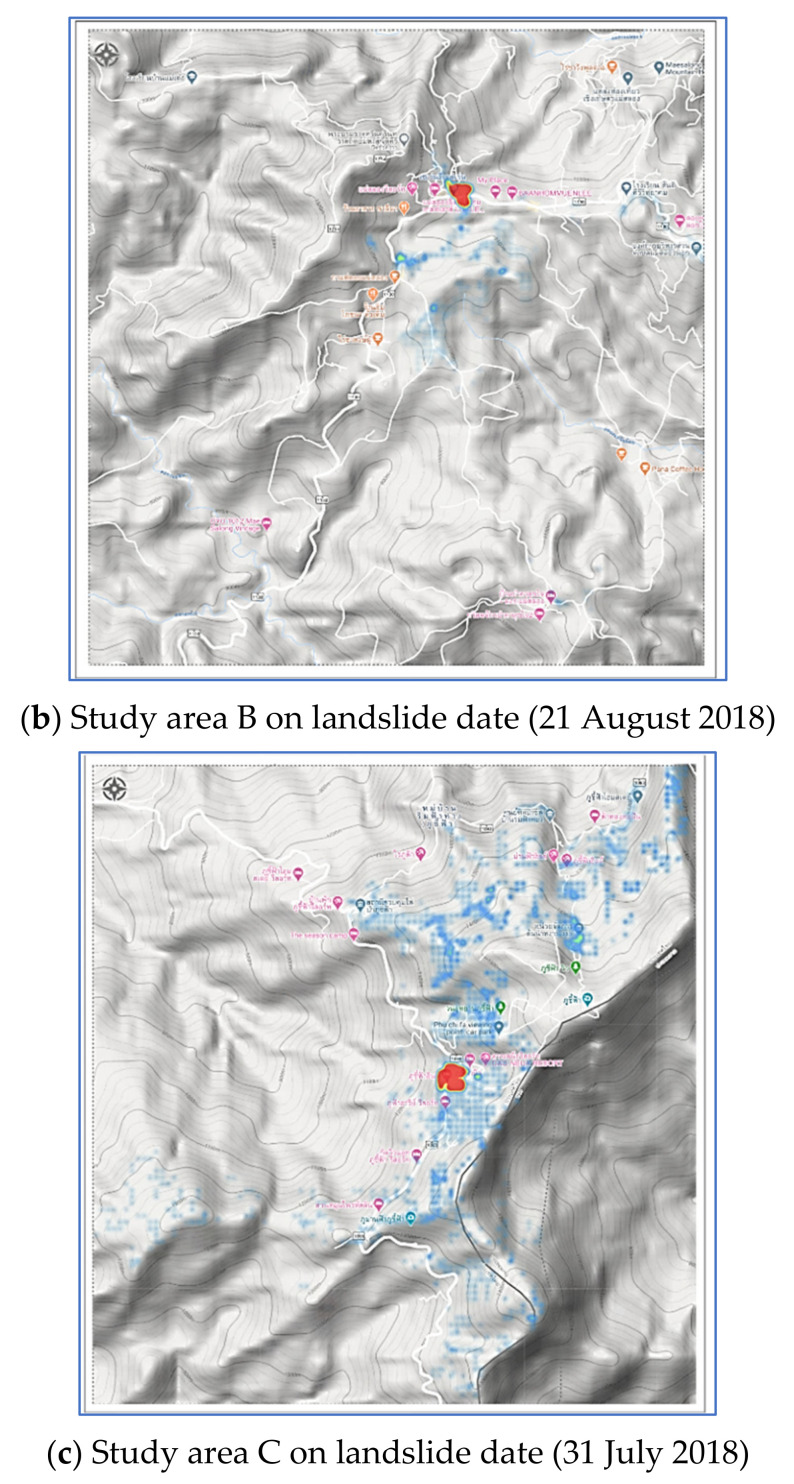
Landslide-risk visualization on Google Maps of Study area A (**a**), B (**b**), and C (**c**).

A well-learned model has been integrated into the website system to create a map-based landslide-risk website. Landslide-risk visualization websites can provide automated, daily updated landslide surveillance through the synergies of various components in the web system by using software tools, i.e., HTML, JavaScript, React, Python, and Python libraries. JavaScript handles interfacing of multiple components in both backend and frontend web processing. The web GIS was deployed on Google Cloud and can be accessed on the landslide-risk website via this link: https://landslide-chiangrai.net (accessed on 30 May 2021). The landslide-risk website presents spatial landslide-risk visualization of the individual study areas i.e., A, B, and C via Google Maps API. Landslide-risk was categorized into 5 levels: negligible risk, very low risk, low risk, medium risk, high risk, and very high risk, corresponding to the estimated landslide-risk probability as in the range of 0.00–0.10, 0.11–0.30, 0.31–0.50, 0.51–0.70, 0.71–0.90, and 0.91–1.0, respectively. Each level is represented on Google Maps by color: white, blue, green, yellow, orange, and red, from negligible risk to very high risk, respectively. The landslide-risk webpage is shown in Figure 29. Examples of GIS map of each study area are presented in Figure 33 and Figure 34.

The latitude and longitude locations, along with the landslide-risk values of all grid cells, have been sent to the Google Maps API for display on the map.

## 4. Conclusions

In this paper, well-known model-based learning methods were conducted for predicting landslide-risk in Chiang Rai, Thailand. Five landslide factors were formulated via QGIS software tool given by Aerial digital images, Google Satellite, and site surveys. The 5 landslide factors were combined with 3 historical landslide occurrences corresponding to 3 Study Areas, exporting CSV files as the landslide dataset via QGIS. The 5 model-based learning methods, LR, ANN, GRU, LSTM, and Bi-LSTM, were evaluated by their predictive performance. Each model determined its optimal parameters given by the set of testing parameters. Enhanced predictive performance using two-stage classifiers—Bi-LSTM combined with Random Forest—were investigated. The best predictive model was selected and combined with variety software components to build the automated landslide-risk web GIS. The experimental results unveil that the 2-stage Bi-LSTM with RF yields the best learning method that is capable of extracting the landslide pattern of the small landslide dataset. The advantages of the proposed landslide-risk web application are: (1) the geographic software tool i.e., QGIS can merge diversity geographical sources into the landslide dataset. The QGIS has user interface that is easy for users to work with. (2) In the case of limited data of historical landslides and few landslides-related factors, the 2-stage Bi-LSTM-RF method is appropriate to create a model for forecasting landslides as demonstrated by the ROC performance. (3) The complexity of automated web GIS is simplified by employed APIs interfacing to other software components of the web application. This has led to increasing the speed of the web development process. (4) The proposed landslide web algorithm can be regarded as a platform for implementing a landslide-risk web GIS in other areas.

## Data Availability

The data presented in this study are available on request from the corresponding author.
